# Breast satisfaction and health-related quality of life following total mastectomy, breast-conserving surgery, or immediate breast reconstruction in Japanese patients with breast cancer: multicentre cross-sectional controlled study (Reborn)

**DOI:** 10.1093/bjsopen/zraf094

**Published:** 2025-09-11

**Authors:** Hirohito Seki, Takako Komiya, Yoshihiro Sowa, Maho Kato, Yutaka Nishida, Hirotsugu Isaka, Jyunji Takano, Shigeru Imoto, Miho Saiga

**Affiliations:** Department of Breast Surgery, Kyorin University School of Medicine, Tokyo, Japan; Department of Plastic and Reconstructive Surgery, Tokyo Medical University, Tokyo, Japan; Department of Plastic Surgery, Jichi Medical University, Tochigi, Japan; Department of Plastic Surgery, Aichi Medical University Hospital, Aichi, Japan; Division of Plastic Surgery, Kamiiida Daiichi General Hospital, Aichi, Japan; Center for Data Science Education and Research, Kyorin University, Tokyo, Japan; Department of Breast Surgery, Kyorin University School of Medicine, Tokyo, Japan; Department of Plastic Surgery, Saitama Medical Center, Saitama, Japan; Department of Breast Surgery, Kyorin University School of Medicine, Tokyo, Japan; Department of Plastic Surgery, Okayama University Hospital, Okayama, Japan

**Keywords:** BREAST-Q, patent-reported outcome, satisfaction with breasts, physical well-being, psychosocial well-being

## Abstract

**Background:**

Surgical decision-making for breast cancer requires consideration of both treatment outcomes and health-related quality of life (HR-QoL). However, data on HR-QoL differences across surgical procedures remain limited. This study compared breast satisfaction and HR-QoL among Japanese patients with breast cancer undergoing mastectomy (MT), breast-conserving surgery (BCS), or immediate breast reconstruction (IBR).

**Methods:**

A survey using the Japanese version of the BREAST-Q was conducted among patients with primary breast cancer who underwent surgery between August 2013 and July 2021.

**Results:**

Of 648 patients, 577 were included in this study. The median time from surgery to questionnaire completion was 56 months. Satisfaction with breast scores was highest in patients undergoing BCS, followed by those undergoing IBR and MT. Psychosocial and sexual well-being were significantly better in patients undergoing BCS and IBR than in those undergoing MT, whereas physical well-being showed no significant differences among the three groups. In multiple regression analysis, surgical procedure was identified as the most influential factor for breast satisfaction, psychosocial well-being, and sexual well-being.

**Conclusions:**

This multicentre Japanese study confirmed that the choice of surgical procedure is the most influential factor affecting postoperative HR-QoL, with both BCS and IBR offering advantages over MT. The findings highlight the importance of comprehensive preoperative counselling to ensure patients receive detailed information on potential HR-QoL differences.

## Teaser

This multicentre, cross-sectional study evaluated patient-reported outcomes using the BREAST-Q to compare breast satisfaction and health-related quality of life among Japanese patients who underwent mastectomy, breast-conserving surgery (BCS), or immediate breast reconstruction (IBR) for breast cancer. Breast satisfaction and psychosocial and sexual well-being were significantly higher in the BCS and IBR groups than in the mastectomy group. Surgical procedure was the most influential factor, underscoring the importance of shared decision-making in optimizing patient-centred care.

## Introduction

Advances in treatment have considerably improved the prognosis of patients with early-stage breast cancer, leading to a paradigm shift in surgical management. Historically, mastectomy (MT) was the only surgical option; however, there has been a gradual increase in the adoption of breast-conserving surgery (BCS) combined with radiation therapy as standard of care^1,2^. In recent years, advances in imaging technology have facilitated the detection of extensive intraductal lesions and multicentric, multifocal disease, whereas improvements in immediate breast reconstruction (IBR) techniques have contributed to a growing trend towards the use of MT^3^. Moreover, contemporary breast cancer surgery requires a balanced approach that achieves both oncological safety and optimal aesthetic functional outcomes^4^.

Previous study have demonstrated improved overall and breast cancer-specific survival in patients undergoing BCS with radiation compared with those undergoing MT alone^5^ . However, concerns have been raised regarding reductions in breast volume that lead to breast asymmetry and the potential deterioration of health-related quality of life (HR-QoL) due to radiation therapy^6^. Although total MT may induce psychological distress because of complete breast loss, it is a therapeutic approach that eliminates the need for radiation therapy associated with BCS^7^. Furthermore, MT avoids the increased risk of complications and multiple surgical procedures often required for breast reconstruction^8^. IBR has become a standard approach for women undergoing MT who wish to maintain their breast shape. The increasing adoption of IBR is attributed to its superior cosmetic outcomes and positive effect on quality of life^9^, with low local recurrence rates and favourable prognosis after local recurrence^10,11^. Furthermore, advances in minimally invasive surgical techniques for IBR and predictive models for assessing the risk of residual tumour have improved the oncological safety of IBR and aesthetic outcomes^12–14^.

In recent years, shared decision-making between patients and healthcare providers has been increasingly recognized as a crucial component in determining treatment strategies^15^. The decision-making process for breast cancer surgery requires information on both treatment-related and HR-QoL outcomes, including physical, psychological, and sexual well-being; changes in body image; and impact on daily life after the procedure^16^. Although traditional outcome evaluations have predominantly relied on subjective assessments by healthcare providers or medical indicators, understanding patients’ perception, evaluation, and values regarding postoperative results is essential for determining appropriate treatment strategies. Patient-reported outcomes (PROs), which reflect HR-QoL and patient satisfaction, are crucial for assessing breast surgery outcomes^17^. The BREAST-Q is a PRO measure designed to assess the impact of breast surgery on patients’ HR-QoL and satisfaction levels. Because of its high reliability and validity, BREAST-Q has been widely adopted globally^18^.

There are numerous comparative studies of postoperative breast satisfaction and HR-QoL between groups undergoing different surgical procedures^19,20^. However, comprehensive studies on differences in satisfaction and HR-QoL among the three surgical approaches (MT, BCS, and IBR) are limited^21^. Furthermore, HR-QoL may be influenced by racial and cultural factors^22^. The aim of the present multicentre cross-sectional study was to elucidate the differences in breast satisfaction and HR-QoL using PROs among Japanese patients with breast cancer who underwent MT, BCS, or IBR.

## Methods

### Study design

This study was conducted at six medical institutions. The participating institutions are facilities certified by the Japanese Breast Cancer Society and consist of university and regional hospitals capable of performing MT, BCS, and IBRs, as well as administering radiation therapy. This study was approved by the Ethics Committee of the lead institution, Kyorin University School of Medicine (IRB no. R05-015), and the institutional review boards of each participating research institution. The data were collected between October 2022 and March 2024.

To be eligible for inclusion in the study, patients had to meet the following criteria: Japanese women with primary breast cancer who underwent surgery between August 2013 and July 2021; had undergone surgery at least 18 months before the survey; age ≥ 20 years at diagnosis; and stage 0–III breast cancer. Women with bilateral breast cancer, secondary breast reconstruction, and metastatic or recurrent breast cancer were excluded from the study, as were those undergoing cosmetic surgery of the contralateral breast or receiving treatment for other malignant diseases, and those who were pregnant. In consideration of the reported improvement in HR-QoL within the first postoperative year, the duration required to complete adjuvant chemotherapy or radiotherapy, and the typical timing of second-stage reconstruction, the questionnaire survey was conducted among patients who had undergone surgery at least 18 months previously^23,24^.

The Japanese version of the BREAST-Q and additional questions on social background were used^25^, with the following subdomains included: satisfaction with breasts, physical well-being (chest), psychosocial well-being, and sexual well-being (*Appendices S1–S8*). BREAST-Q, authored by Drs Klassen, Pusic and Cano, was used under license from Memorial Sloan Kettering Cancer Center (New York, NY, USA). The social background section included items on education level, employment status, the presence of a partner, participation in skin-exposing activities (for example, swimming and hot spring use), and engagement in physical activities (for example, various sports activities and dance). Patients were asked to answer approximately 50 questions using a paper-based questionnaire, which required about 10 minutes to complete. All completed questionnaires were mailed in sealed envelopes to the data centre (*Fig. S1*). Patients’ clinical information was extracted from their medical records and collected at the data centre in an electronic format.

### Required sample size

Information from previous research was used to calculate the sample size, with a minimal important difference defined as 4 points^21,26^. When conducting a one-way analysis of variance (ANOVA) with three groups having equal sample sizes and a standard deviation of 20 in each group, the effect size (*f*) was 0.163. The required sample size to detect a 4-point difference was determined to be 122 patients per group (using G*Power 3.1.9.7 parameters as follows: type I error probability α = 0.05, power = 0.80, and effect size *f* = 0.163).

### Statistical analysis

Categorical variables among the three groups were compared using the χ^2^ test. Continuous variables are presented as the median with interquartile range (i.q.r.) or as a mean with the 95% confidence interval (c.i.). For multiple comparisons, mean values were compared using one-way ANOVA or unpaired *t* tests. Tukey's honestly significant difference test and the Bonferroni method were applied as post hoc tests. Multiple regression analysis was used for multivariate analysis.

All statistical analyses were performed using SPSS^®^ version 29.0 (IBM, Armonk, NY, USA). Statistical significance was set at two-tailed *P* < 0.05. In multiple comparisons, significance was determined based on adjusted *P* values.

## Results

### Patient inclusion

**Fig. 1:**
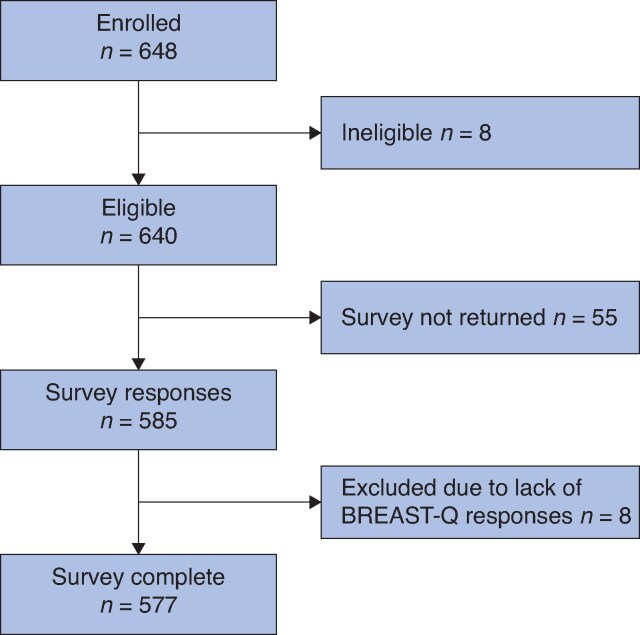
Flow chart showing patient recruitment The survey response rate was 90.2% (577 of 640).

Of the 648 patients initially enrolled in this study, 8 were deemed ineligible, 55 did not respond to requests to take part in the study, and 8 did not complete the BREAST-Q, and were therefore excluded. Thus, 577 patients were included in the final analysis. The survey response rate was 90.2% (577 of 640) (*Fig. 1*).

### Patient characteristics

The median time from surgery to questionnaire response was 52 months (*Table 1*). The median age of patients at the time of diagnosis was 58, 53, and 48 years in the MT, BCS, and IBR groups, respectively (*P* < 0.001). The MT group had a significantly higher prevalence of patients with clinical T3 or T4 status, clinically positive axillary lymph node metastasis (N), axillary lymph node dissection, and chemotherapy than the other groups. Of the patients in the BCS group, 88.1% received postoperative radiation therapy. Clavien–Dindo grade ≥ III complications^27^ were significantly more frequent in the IBR group than in the MT and BCS groups (*P* < 0.001). Among the patients in the IBR group, 39.9% underwent nipple-sparing MT and 72.2% underwent implant-based reconstruction. With regard to employment status, the proportion of full-time employees was significantly higher in the BCS and IBR groups than in the MT group (*P* < 0.001). The frequency of skin-exposing activities (for example, swimming and hot spring use) was also significantly higher in the BCS and IBR groups than in the MT group (*P* = 0.003) (*Table S1*).

**Table 1:** Patient characteristics (clinical background)

	All patients (*n* = 577)	MT (*n* = 194)	BCS (*n* = 185)	IBR (*n* = 198)	P*
Time from surgery to questionnaire response (months), median (i.q.r.)	52.0 (32–75.75)	53.0 (32.0–76.0)	44.0 (29.0–72.25)	57.0 (34.0–76.0)	0.406
Age at diagnosis (years), median (i.q.r.)	51 (45–61)	58 (49–67)	54 (46–63)	48 (43–52)	<0.001
BMI (kg/m^2^), median (i.q.r.)	21.6 (19.6–24.5)	21.9 (19.6–24.5)	22.0 (19.8–25.8)	21.3 (19.4–23.8)	0.108
**Menopausal status**					<0.001
Premenopausal	273 (47.3%)	67 (34.5%)	85 (45.9%)	121 (61.1%)	
Postmenopausal	274 (47.5%)	124 (63.9%)	98 (53.0%)	52 (26.3%)	
Unknown	30 (5.2%)	3 (1.5%)	2 (1.1%)	25 (12.6%)	
**Smoking**					<0.001
No	510 (88.4%)	182 (93.8%)	170 (91.9%)	158 (79.8%)	
Yes	48 (8.3%)	10 (5.2%)	7 (3.8%)	31 (15.7%)	
Unknown	19 (3.3%)	2 (1.0%)	8 (4.3%)	9 (4.5%)	
**Primary tumour size (T)**					
Is	98 (17.0%)	18 (9.3%)	8 (4.3%)	72 (36.4%)	<0.001
1	287 (49.7%)	83 (42.8%)	133 (71.9%)	71 (35.9%)	
2	159 (27.6%)	70 (36.1%)	41 (22.2%)	48 (24.2%)	
3	5 (0.9%)	4 (2.1%)	0 (0%)	1 (0.5%)	
4	21 (3.6%)	18 (9.3%)	2 (1.1%)	1 (0.5%)	
Unknown	7 (1.2%)	1 (0.5%)	1 (0.5%)	5 (2.5%)	
**Clinical node status (N)**					
0	488 (84.6%)	152 (78.4%)	169 (91.4%)	167 (84.3%)	0.003
1	71 (12.3%)	34 (17.5%)	14 (7.6%)	23 (11.6%)	
2	7 (1.2%)	5 (2.6%)	1 (0.5%)	1 (0.5%)	
3	5 (0.9%)	3 (1.5%)	0 (0%)	2 (1.0%)	
Unknown	6 (1.0%)	0 (0%)	1 (0.5%)	5 (2.5%)	
**Axillary surgery**					<0.001
SLNB	483 (83.7%)	144 (74.2%)	172 (93.0%)	167 (83.7%)	
ALND	82 (14.2%)	46 (23.7%)	8 (4.3%)	28 (14.1%)	
None	6 (1.0%)	1 (0.5%)	5 (2.7%)	0 (0%)	
Unknown	6 (1.0%)	3 (1.5%)	0 (0%)	3 (1.5%)	
**Chemotherapy**					<0.001
No	405 (70.2%)	115 (59.3%)	149 (80.5%)	141 (71.2%)	
Yes	169 (29.3%)	79 (40.7%)	36 (19.5%)	54 (27.3%)	
Unknown	3 (0.5%)	0 (0%)	0 (0%)	3 (1.5%)	
**Postoperative radiation therapy**					<0.001
No	364 (63.1%)	166 (85.6%)	22 (11.9%)	176 (88.9%)	
Yes	213 (36.9%)	28 (14.4%)	163 (88.1%)	22 (11.1%)	
**Complications (Clavien–Dindo grade ≥ III)**					<0.001
No	560 (97.1%)	191 (98.5%)	185 (100%)	184 (97.1%)	
Yes	17 (2.9%)	3 (1.5%)	0 (0%)	14 (7.1%)	
**Mastectomy procedure (IBR only)**					
NSM				79 (39.9%)	
SSM/MT				119 (60.1%)	
**Reconstruction method (IBR only)**					
Implant				143 (72.2%)	
Autologous				55 (27.7%)	
**Reconstruction timing (IBR only)**					
Immediate one-stage				43 (21.7%)	
Immediate two-stage				155 (78.3%)	

Values are *n* (%) unless otherwise stated. MT, total mastectomy; BCS, breast-conserving surgery; IBR, immediate breast reconstruction; i.q.r., interquartile range; BMI, body mass index; SLNB, sentinel lymph node biopsy; ALND, axillary lymph node dissection; NSM, nipple-sparing mastectomy; SSM, skin-sparing mastectomy. *Categorical variables among the three groups were compared using the χ2 test.

### BREAST-Q scores in the MT, BCS, and IBR groups

**Fig. 2:**
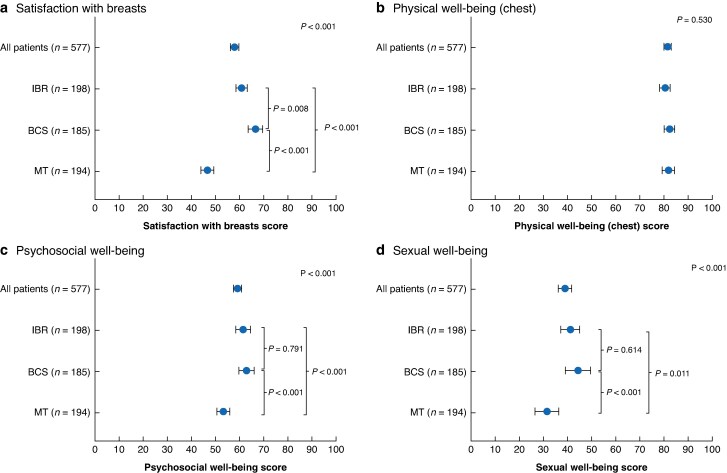
Scores for different domains on the BREAST-Q in all patients and according to type of procedure **a** Satisfaction with breasts. **b** Physical well-being (chest). **c** Psychosocial well-being. **d** Sexual well-being. Symbols show mean values, with error bars indicating 95% confidence intervals. MT, total mastectomy; BCS, breast-conserving surgery; IBR, immediate breast reconstruction.

The BCS group had the highest score for the BREAST-Q Satisfaction with breast domain (mean 66.7; 95% c.i. 63.7 to 69.8), followed by the IBR and MT groups (60.9 (95% c.i. 58.6 to 63.3) and 46.7 (95% c.i. 44.1 to 49.4), respectively), with a significant difference among the three groups (*P* < 0.001) (*Fig. 2a*). Physical well-being (chest) scores did not differ significantly among the three groups (*P* = 0.530) (*Fig. 2b*). However, there were significant differences among the MT, BCS, and IBR groups in psychosocial well-being scores (mean 53.3 (95% c.i. 50.6 to 56.0), 62.9 (95% c.i. 59.7 to 66.1), and 61.5 (95% c.i. 58.5 to 64.5), respectively; *P* < 0.001) (*Fig. 2c*) and sexual well-being scores (mean 31.6 (95% c.i. 26.8 to 36.5), 44.5 (95% c.i. 39.2 to 49.8), and 41.3 (95% c.i. 37.4 to 45.3), respectively; *P* < 0.001) (*Fig. 2d*). In the comparisons of BREAST-Q domains between implant-based and autologous reconstruction, a significant difference was observed only in the satisfaction with breast domain score (mean 59.2 *versus* 65.6, respectively) (*Table S2*). A comparison of BREAST-Q domains based on the presence or absence of nipple–areola complex preservation in the IBR group showed a significant difference in physical well-being domain scores (mean 82.7 *versus* 77.8 for skin-sparing MT/MT *versus* nipple-sparing mastectomy, respectively; *P* = 0.031) (*Table S3*).

### Multiple regression analysis of BREAST-Q domains

The associations between each BREAST-Q domain and clinical and social factors were evaluated using multiple regression analysis. Surgical procedure was identified as the most influential factor for satisfaction with breasts (MT *versus* BCS: β = 0.471; MT *versus* IBR: β = 0.287), psychosocial well-being (MT *versus* BCS: β = 0.244; MT *versus* IBR: β = 0.127), and sexual well-being (MT *versus* BCS: β = 0.276; MT *versus* IBR: β = 0.166) (*Table 2*).

**Table 2:** Multiple regression analysis of each BREAST-Q domain

	Satisfaction with breasts		Physical well-being		Psychosocial well-being		Sexual well-being	
	β	*P*	β	*P*	β	*P*	β	*P*
**Surgical procedure**								
MT *versus* BCS	0.471	<0.001	−0.009	0.905	0.244	0.001	0.276	0.009
MT *versus* IBR	0.287	<0.001	−0.028	0.618	0.127	0.021	0.166	0.040
Postoperative period	−0.044	0.279	0.147	0.001	0.009	0.836	−0.121	0.055
Age at surgery	−0.146	0.002	0.010	0.847	−0.098	0.050	−0.142	0.043
Axillary surgery	0.019	0.698	−0.026	0.631	0.027	0.607	0.085	0.265
Chemotherapy	−0.023	0.620	0.053	0.308	0.094	0.062	0.035	0.630
Radiation therapy	−0.039	0.542	0.008	0.912	−0.074	0.276	0.005	0.958
Complications	−0.026	0.522	−0.050	0.268	−0.067	0.129	−0.018	0.773
Education level	−0.004	0.925	0.016	0.727	0.001	0.974	−0.090	0.159
Employment status	0.031	0.480	0.008	0.876	−0.053	0.260	−0.007	0.911
Has a partner	0.028	0.505	0.021	0.652	−0.071	0.109	−0.060	0.349
Skin-exposing activities*	0.068	0.102	−0.034	0.461	0.025	0.573	0.107	0.098
Physically active†	0.036	0.382	0.082	0.072	0.117	0.008	0.085	0.177

*Skin-exposing activities such as swimming and hot spring use. †Engages in physical activities such as sports activities and dance. β, standardized coefficient; MT, total mastectomy; BCS, breast-conserving surgery; IBR, immediate breast reconstruction.

## Discussion

Although the expanded range of treatment options has enabled more flexible approaches, determining the optimal surgical procedure for each patient remains a critical challenge because each procedure has its own advantages and disadvantages. In this study, breast aesthetic satisfaction was significantly higher in the BCS group than in the MT and IBR groups, whereas psychosocial and sexual well-being were significantly better in the IBR and BCS groups than in the MT group. The type of surgical procedure was the most influential factor on BREAST-Q scores. However, the findings of this study do not definitively establish the superiority of any one surgical approach over the others, which highlights the importance of comprehensive preoperative counselling to ensure that patients receive detailed information on potential HR-QoL differences. The findings of this study may contribute to more appropriate decision-making by patients with breast cancer.

The finding that breast satisfaction was significantly lower in the MT group than in the BCS and IBR groups is consistent with findings reported previously^21,28^. In addition, breast satisfaction in the present study was significantly higher in the BCS group than in the IBR group, which contrasts with the findings reported by Howes *et al*.^21^. Several factors could explain this discrepancy, such as the time interval between surgery and survey responses, which is known to influence breast satisfaction and HR-QoL in patients who underwent BCS^29–31^. In the present study, the median time from surgery to survey completion was approximately 56 months in both groups. In contrast, Howes *et al*.^21^ reported that the median postoperative period was significantly shorter in the BCS group (29 months) than in the IBR group (48 months), which may have influenced their results.

In addition, breast asymmetry may have played a role in breast satisfaction scores in the BCS group, with Howes *et al*.^21^ reporting that 75.3% of women who underwent BCS perceived some degree of breast asymmetry and that 15.5% of these women considered the asymmetry sufficiently problematic to consider surgical correction. Cochrane *et al*.^32^ reported that the proportion of excised breast volume was a critical determinant of cosmetic outcomes and patient satisfaction after BCS. Oncoplastic BCS results in superior aesthetic outcomes and higher patient satisfaction than the standard BCS^33^. Although it is unclear how many patients in the BCS group in the present study underwent oncoplastic BCS, this technique has been increasingly adopted in Japan since the early 2010s, potentially contributing to the higher breast satisfaction observed in the BCS group^34^.

Differences in reconstruction methods may have also influenced the results. In a previous study^21^, approximately 60% of patients underwent autologous tissue reconstruction, whereas in the present study this proportion was approximately 30%. Patients who undergo autologous tissue reconstruction report higher satisfaction with their reconstructed breasts than those who undergo implant-based reconstruction^35–37^, with these findings consistent across multiple studies using the BREAST-Q. The relatively low proportion of patients undergoing autologous reconstruction in the present study may have contributed to the lower breast satisfaction observed in the IBR group. Studies comparing BCS with MT and reconstruction have shown that BCS leads to higher patient satisfaction and better body image outcomes^38,39^. Patients who undergo BCS have been reported to have significantly higher breast satisfaction scores than those who undergo MT with implant reconstruction^38,39^. BCS is also associated with better HR-QoL scores across various subscales^38^. Although one study^40^ found no significant difference in body image between the BCS and reconstruction groups, patients undergoing BCS reported higher satisfaction with surgery. Notably, patients with free-flap reconstruction had satisfaction scores comparable to those who underwent BCS, whereas those with implant-based reconstructions scored lower^39^. These findings suggest that BCS may be preferable when oncologically appropriate because it offers better PROs. On the basis of the results of the present study alone, it cannot be definitively concluded that BCS results in higher breast satisfaction than IBR. However, it is unequivocal that breast satisfaction in both the BCS and IBR groups is superior to that in the MT group.

Howes *et al*.^21^ reported that patients who underwent BCS experienced a greater decline in physical well-being than those who underwent MT or IBR; however, in the present study, there was no significant difference in physical well-being among the three groups. This discrepancy may again be attributed to the relatively shorter time interval between surgery and survey completion in the BCS group than in the other two groups in the previous study^21^. One possible factor contributing to the decline in physical well-being after BCS is the impact of postoperative radiotherapy. However, patients undergoing BCS who receive postoperative radiotherapy generally experience only mild and temporary impairments^41^. Furthermore, studies examining the effects of radiotherapy on the HR-QoL of patients with breast cancer undergoing BCS suggest that the long-term effects are minimal^41,42^. Zehra *et al*.^28^ reported that the BCS and IBR groups had better outcomes in terms of physical well-being than the MT alone group. In addition, Warm *et al*.^43^ found no significant differences in the well-being or quality of life of patients based on the type of surgery performed. These conflicting reports suggest that the effect of surgical approaches on physical well-being varies among individuals and may change over time. This underscores the importance of personalized treatment decisions and the need for long-term follow-up to comprehensively assess patient outcomes.

In this study, psychosocial well-being was significantly higher in the BCS and IBR groups than in the MT group. Howes *et al*.^21^ reported no differences among the three surgical approaches and Zehra *et al*.^28^ found that breast reconstruction yielded better outcomes in terms of body image than MT alone. Similarly, Vohra *et al*.^44^ observed that patients who underwent MT with reconstruction had higher psychosocial well-being scores than those who underwent MT alone. In addition, Moyer *et al*.^45^ reported that BCS conferred slight advantages in terms of psychological well-being, marital intimacy, social adaptation, body image, and cancer-related concerns. Together, these findings suggest that both breast reconstruction and BCS may offer psychosocial benefits over MT alone.

Although satisfaction with breast aesthetics and HR-QoL may be influenced by various factors beyond surgery, the findings of the present study confirmed that the type of surgical procedure was the most influential factor. It has been reported that surgery type is an independent predictor of breast satisfaction and psychosocial well-being^46^. Although age was also identified as a factor contributing to breast satisfaction and sexual well-being in the present study, the age distribution in the IBR group may have influenced these findings. Higher levels of physical activity may positively influence the psychosocial well-being of patients^47^ .

In the present study, the potential presence of selection and recall biases is a concern. However, the findings hold academic significance as the first multicentre cross-sectional study in Japan, conducted within this unique social context, characterized by racial homogeneity among patients and a relatively uniform healthcare system. In the future, prospective studies evaluating breast satisfaction and HR-QoL in patients with breast cancer, as well as research on decision-making processes, are warranted.

## Supplementary Material

zraf094_Supplementary_Data

## Data Availability

The data sets are not publicly available due to restrictions imposed under the license for the present study, but are available from the corresponding author upon reasonable request.
