# Supplementary material for: Breast satisfaction and health-related quality of life following total mastectomy, breast-conserving surgery, or immediate breast reconstruction in Japanese patients with breast cancer: multicentre cross-sectional controlled study (Reborn)
Source: BJS Open. 2025 Sep 11;9(5):zraf094. doi: 10.1093/bjsopen/zraf094 (PMC12461568; doi:10.1093/bjsopen/zraf094)
Supplement: zraf094_Supplementary_Data [file zraf094_supplementary_data.docx]

**Title**

**Breast satisfaction and health-related quality of life following total mastectomy, breast-conserving surgery, and immediate breast reconstruction in Japanese patients with breast cancer: multicentre cross-sectional controlled study (Reborn)**

Authors

Hirohito Seki, MD, PhD^1^, Takako Komiya, MD, PhD^2^, Yoshihiro Sowa, MD, PhD ^3^, Maho Kato, MD, PhD^4,5^, Yutaka Nisida, PhD ^6^, Hirotsugu Isaka, MD^1^, Jyunji Takano, MD, PhD ^6^, Shigeru Imoto, MD, PhD ^1^, Miho Saiga, MD, PhD ^8^

On behalf of Collaborative Study Group of Scientific Research of the Japan Oncoplastic Breast Surgery Society

1. Department of Breast Surgery, Kyorin University School of Medicine, Tokyo, Japan
2. Department of Plastic and Reconstructive Surgery, Tokyo Medical University Tokyo, Japan
3. Department of Plastic Surgery, Jichi Medical University, Tochigi, Japan
4. Department of Plastic Surgery, Aichi Medical University Hospital Aichi, Japan
5. Division of Plastic Surgery, Kamiiida Daiichi General Hospital Aichi, Japan
6. Center for Data Science Education and Research, Kyorin University Tokyo, Japan
7. Department of Plastic Surgery, Saitama Medical Center, Saitama, Japan
8. Department of Plastic Surgery, Okayama University Hospital, Okayama, Japan

***Corresponding author:** Hirohito Seki, MD, PhD

Department of Breast Surgery, Kyorin University School of Medicine

6-20-2 Shinkawa, Mitaka-city, Tokyo, 181-8611, Japan

Tel: +81-422-47-5511 Ext 7876; Fax: +81-422-44-0751

Email: [hirohito-seki@ks.kyorin-u.ac.jp](mailto:hirohito-seki@ks.kyorin-u.ac.jp)

ORCID ID: 0000-0002-3211-4817

**Supplementary Materials - Index**

| **Supplementary Appendixes** |  |
| --- | --- |
| **Supplementary Appendix 1: Satisfaction with breasts (questionnaire)** | *page 3* |
| **Supplementary Appendix 2: Satisfaction with breasts (conversion table)** | *page 4* |
| **Supplementary Appendix 3: Physical well-being: chest (questionnaire)** | *page 5* |
| **Supplementary Appendix 4: Physical well-being: chest (conversion table)** | *page 6* |
| **Supplementary Appendix 5: Psychosocial well-being (questionnaire)** | *page 7* |
| **Supplementary Appendix 6: Psychosocial well-being (conversion table)** | *page 8* |
| **Supplementary Appendix 7: sexual well-being (questionnaire)** | *page 9* |
| **Supplementary Appendix 8: sexual well-being (conversion table)** | *page 10* |
| **Supplementary Figures and Tables** |  |
| **Supplementary Figure 1 Study diagram** | *page 11* |
| **Supplementary Table 1. Patients' characteristics (social backgrounds)** | *page 12-13* |
| **Supplementary Table2. Comparison of breast satisfaction and HR-QOL between implant-based and autologous tissue reconstruction** | *page 14* |
|  |  |

**Supplementary Appendixes**

**Supplementary Appendix 1: Satisfaction with breasts (questionnaire)**

**Supplementary Appendix 2: Satisfaction with breasts (conversion table)**

**Supplementary Appendix 3: Physical well-being: chest (questionnaire)**

**Supplementary Appendix 4: Physical well-being: chest (conversion table)**

**Supplementary Appendix 5: Psychosocial well-being (questionnaire)**

**Supplementary Appendix 6: Psychosocial well-being (conversion table)**

**Supplementary Appendix 7: sexual well-being (questionnaire)**

**Supplementary Appendix 8: sexual well-being (conversion table)**

**Supplementary Figures and Tables**

**Supplementary Figure 1 Study diagram**

**
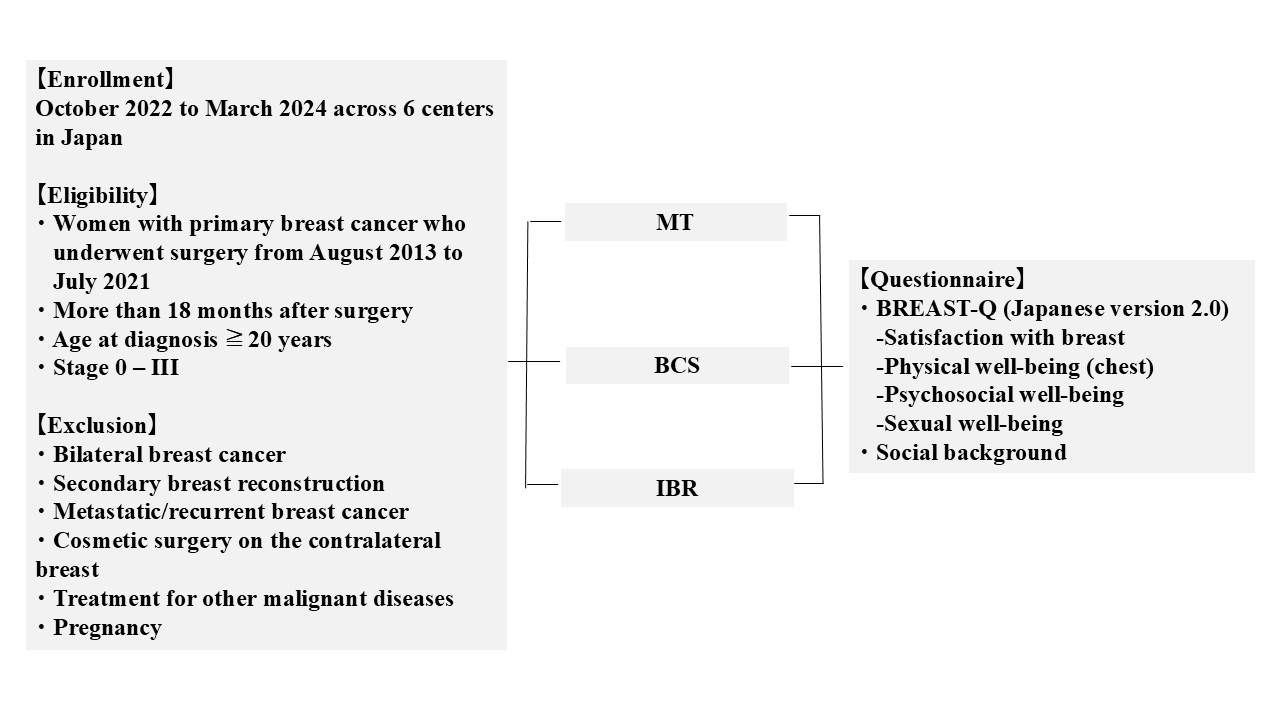
**

MT, total mastectomy; BCS, breast-conserving surgery; IBR, immediate breast reconstruction.

**Supplementary Table 1. Patients' characteristics (social backgrounds)**

| Variable | Total  (n = 577) | MT  (n = 194) | BCS  (n = 185) | IBR  (n = 198) | P-value |
| --- | --- | --- | --- | --- | --- |
| Education level |  |  |  |  | 0.372 |
| Junior high school/high school/ vocational school/junior college | 408 (70.7%) | 139 (71.6%) | 134 (72.4%) | 135 (68.2%) |  |
| University | 143 (24.8%) | 43 (22.2%) | 43 (23.2%) | 57 (28.8%) |  |
| Graduate School | 6 (1.0%) | 4 (2.1%) | 0 (0%) | 2 (1.0%) |  |
| Prefer not to answer | 14 (2.4%) | 6 (3.1%) | 6 (3.2%) | 2 (1.0%) |  |
| Not answered | 6 (1.0%) | 2 (1.0%) | 2 (1.1%) | 2 (1.0%) |  |
| Employment status |  |  |  |  | <0.001 |
| Full-time employee | 174 (30.2%) | 34 (17.5%) | 55 (29.7%) | 85 (30.2%) |  |
| Part-time employee | 169 (29.3%) | 57 (29.4%) | 58 (31.4%) | 54 (27.3%) |  |
| Unemployed | 208 (36.0%) | 90 (46.4%) | 63 (34.1%) | 55 (27.8%) |  |
| Prefer not to answer | 20 (3.5%) | 11 (5.7%) | 7 (3.8%) | 2 (1.0%) |  |
| Not answered | 6 (1.0%) | 2 (1.0%) | 2 (1.1%) | 2 (1.0%) |  |
| Presence of partner |  |  |  |  | 0.287 |
| No | 124 (21.5%) | 52 (26.8%) | 31 (16.8%) | 41 (20.7%) |  |
| Yes | 427 (74.0%) | 134 (69.1%) | 143 (77.3%) | 150 (75.8%) |  |
| Prefer not to answer | 16 (2.8%) | 5 (2.6%) | 6 (3.2%) | 5 (2.5%) |  |
| Not answered | 10 (1.7%) | 3 (1.5%) | 5 (2.7%) | 2 (1.0%) |  |
| Skin-exposing activities |  |  |  |  | 0.003 |
| No | 293 (50.8%) | 118 (60.8%) | 87 (48.3%) | 88 (44.7%) |  |
| Yes | 274 (47.5%) | 72 (37.1%) | 93 (51.7%) | 109 (55.3%) |  |
| Not answered | 10 (1.7%) | 4 (2.1%) | 0 (0%) | 0 (0%) |  |
| Physical activities |  |  |  |  | 0.078 |
| No | 381 (66.0%) | 140 (72.2%) | 114 (61.6%) | 127 (64.1%) |  |
| Yes | 186 (32.2%) | 50 (26.3%) | 66 (35.7%) | 70 (35.4%) |  |
| Not answered | 10 (1.8%) | 4 (1.5%) | 5 (2.7%) | 1 (0.5%) |  |

Abbreviations: MT, total mastectomy; BCS, breast-conserving surgery; IBR, immediate breast reconstruction

| **Supplementary Table2. Comparison of breast satisfaction and HR-QOL between implant-based and autologous tissue reconstruction** | | | | | | | | | |
| --- | --- | --- | --- | --- | --- | --- | --- | --- | --- |
|  |  |  |  |  |  |  |  |  |  |
| Surgical procedure | Satisfaction with breasts | | Physical well-being (chest) | | Psychosocial well-being | | Sexual well-being | |  |
|  | Mean (SE) | P-value | Mean (SE) | P-value | Mean (SE) | P-value | Mean (SE) | P-value |  |
| Implant reconstruction | 59.2 (1.385) | 0.017 | 80.7 (1.310) | 0.967 | 61.0 (1.811) | 0.553 | 40.5 (2.287) | 0.490 |  |
| Autologous reconstruction | 65.6 (2.269) |  | 80.8 (2.293) |  | 63.0 (2.762) |  | 44.7 (3.931) |  |  |
|  |  |  |  |  |  |  |  |  |  |
| Abbreviations: SE, standard error | | | | | | |  |  |  |
|  |  |  |  |  |  |  |  |  |  |

| **Supplementary Table3. Comparison of breast satisfaction and HR-QOL based on the presence or absence of nipple-areola complex preservation in immediate breast reconstruction** | | | | | | | | | | | | | |
| --- | --- | --- | --- | --- | --- | --- | --- | --- | --- | --- | --- | --- | --- |
|  |  |  |  |  |  |  |  |  | |  |  |  |  |
| Satisfaction with breasts | | Physical well-being (chest) | | Psychosocial well-being | | Sexual well-being | |  | |  |  |  |  |
| Mean (SE) | P-value | Mean (SE) | P-value | Mean (SE) | P-value | Mean (SE) | P-value |  | |  |  |  |  |
| 61.6 (1.625) | 0.527 | 82.7 (1.526) | 0.031 | 62.6 (1.981) | 0.404 | 42.5 (2.598) | 0.442 |  | |  |  |  |  |
| 60.0 (1.746) |  | 77.8 (1.649) |  | 60.0 (2.350) |  | 39.4 (2.980) |  |  | |  |  |  |  |
|  |  |  |  |  |  |  |  |  | |  |  |  |  |
| Abbreviations: SE, standard error; SSM, skin-sparing mastectomy; MT, mastectomy; NSM, nipple-sparing mastectomy | | | | | | | | |  | | |  |  |
